# 3D Hydrodynamic Focusing in Microscale Optofluidic Channels Formed with a Single Sacrificial Layer

**DOI:** 10.3390/mi11040349

**Published:** 2020-03-27

**Authors:** Erik S. Hamilton, Vahid Ganjalizadeh, Joel G. Wright, Holger Schmidt, Aaron R. Hawkins

**Affiliations:** 1 Electrical and Computer Engineering, Brigham Young University, Provo, UT 84602, USA; joel.g.wright@studentbody.byu.edu (J.G.W.); ahawkins@byu.edu (A.R.H.); 2 Electrical and Computer Engineering, University of California, Santa Cruz, Santa Cruz, CA 95064, USA; vganjali@ucsc.edu (V.G.); hschmidt@soe.ucsc.edu (H.S.)

**Keywords:** 3D hydrodynamic focusing, optofluidic, lab-on-a-chip, biosensor, microscale channel, microfluidic, liquid-core waveguide, single layer, reservoir effect

## Abstract

Optofluidic devices are capable of detecting single molecules, but greater sensitivity and specificity is desired through hydrodynamic focusing (HDF). Three-dimensional (3D) hydrodynamic focusing was implemented in 10-μm scale microchannel cross-sections made with a single sacrificial layer. HDF is achieved using buffer fluid to sheath the sample fluid, requiring four fluid ports to operate by pressure driven flow. A low-pressure chamber, or pit, formed by etching into a substrate, enables volumetric flow ratio-induced focusing at a low flow velocity. The single layer design simplifies surface micromachining and improves device yield by 1.56 times over previous work. The focusing design was integrated with optical waveguides and used in order to analyze fluorescent signals from beads in fluid flow. The implementation of the focusing scheme was found to narrow the distribution of bead velocity and fluorescent signal, giving rise to 33% more consistent signal. Reservoir effects were observed at low operational vacuum pressures and a balance between optofluidic signal variance and intensity was achieved. The implementation of the design in optofluidic sensors will enable higher detection sensitivity and sample specificity.

## 1. Introduction

The optofluidic detection of particles in liquid waveguide channels has recently grown in significance [1,2,3,4,5,6,7]. The intersecting of solid-core waveguides with liquid-core waveguides enables light-matter interaction, such as fluorescence generation for single molecule detection. This is especially interesting for identification of particles in liquid, such as disease pathogens [8,9,10,11,12,13,14]. The small scale of the optofluidic channels is critical for single molecule detection in flow.

The nature of the microfluidic channels found on optofluidic sensor platforms sets some of the ultimate sensitivity and accuracy limits. The small cross-sectional area of the channels typically means operation in the laminar flow regime, which results in a parabolic velocity distribution. Fluid that is in the center of the channel flows faster than fluid at the walls. This means fluorescent particles, being distributed evenly through a channel, would spend different amounts of time in perpendicular excitation light beams as they flow past excitation points introducing variation in excitation times and fluorescent signal generation. Additionally, the excitation light exhibits an optical mode intensity profile that results in a distribution of generated fluorescence intensities. Additionally, the collection efficiency of fluorescence in a liquid-core waveguide is dependent on particle position [15,16]. Particles that are near the center of the channel experience higher collection efficiency. Moreover, velocity-based identification schemes suffer from the particle velocity distribution as variation limits the sensitivity and specificity capabilities of these methods. Taken together, all of these possible variations in signal intensity and particle velocity distribution introduce uncertainty in particle detection.

Hydrodynamic focusing (HDF) promises to enhance the detection capabilities in optofluidic channels by controlling the position of target particles in the fluid channel. This results in narrower velocity and excitation intensity distributions, which means less variation in the fluorescent signal intensity distribution [17]. Three-dimensional hydrodynamic focusing (3DHDF) aims to limit the position of target particles both horizontally and vertically. This ensures they pass through the excitation beam, flow at a more uniform velocity, and experience optimal collection efficiencies resulting in less fluorescent signal intensity variation. We have predicted a signal enhancement of between three and five times with 3DHDF, but the complexity of implementation should be considered.

**Figure 1:**
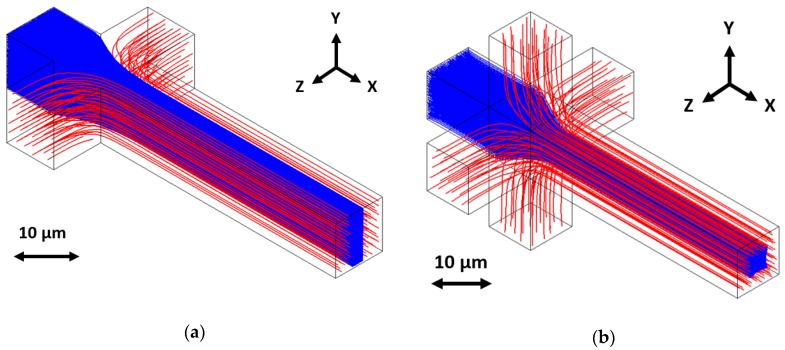
Hydrodynamic focusing occurs when sample fluid (drawn in solid blue) is squeezed and sheathed from (**a**) two dimensions (horizontal focusing) or (**b**) three dimensions (horizontal and vertical focusing) by a buffer fluid (drawn in striped red), controlling the position of sample fluid in the fluid channel.

HDF is commonly achieved using buffer fluid or sheath flows to surround and squeeze a sample fluid stream. The engineering challenge comes in designing and fabricating the intersection for these flows. Two-dimensional hydrodynamic focusing is relatively straightforward, even at the 10-μm scale. It simply requires flowing buffer fluid from the left and right sides of the sample stream to achieve horizontal, or in-plane, focusing, as in Figure 1a. The buffer fluid occupies the edges of the microfluidic channel volume, limiting the locations that sample particles (flowing in from a central channel) can occupy. Focusing the sample stream from the top and bottom, or vertically, is much more difficult to achieve, because the fluid must be directed from out-of-plane. This can lead to complex designs, difficult fabrication processes, and complicated operation. However, the combination of horizontal and vertical focusing resulting in a sample stream surrounded on all sides, or sheathed, by buffer fluid, as in Figure 1b, enables enhanced optofluidic performance and applications [18,19,20,21,22]. Our goal with this work was to develop a simple buffer fluid induced 3D focusing scheme at the 10-μm scale while using surface micromachining. Ultimately, we desired a design only requiring a single sacrificial layer of material to avoid complexity in fabrication and maximize device yield. We also wanted to keep device operation as simple as possible.

A wide variety of interesting designs have been developed to perform 3DHDF while using buffer fluid at the microscale. One existing method literally injects sample fluid into a buffer fluid stream using a nanoneedle, or micronozzle [23]. The micronozzle cantilever is formed with oxide over silicon before the silicon is etched and the nanoneedle released. This fragile structure extends into the 100 µm scale microfluidic channel, where buffer fluid occupies the volume and performs the sheathing function for 3DHDF. Not only is the fabrication complex, but the structure is fragile and it might be impossible to shrink to the 10-μm scale as the oxide annealing process that forms the nozzle may lead to a closed nozzle. Alternatively, tilted lithography has been used to create fluid manifolds for directing buffer fluid around sample fluid for sheathing [24]. The fabrication process requires a rotatable, tiltable jig holder for the microfluidic device during multiple photolithography exposures. The design is similar to the micronozzle, in that it includes a sample injection nozzle. Again, the structures are on the 100 μm scale. They both require three or four fluid reservoirs to operate, depending on layout. Other designs requiring complex fabrication processes or operation include a femtosecond laser exposure design and a PDMS stack that requires five to seven layers to make and six fluid reservoirs to operate [25,26].

Simpler, “single layer” designs make use of clever fluid dynamics properties to induce 3DHDF with buffer fluid through secondary flow production. This takes the form of Dean Vortex generation in fast moving fluids. The vortices induce flow orthogonal to the main flow direction, drawing out and shaping the sample stream. The microscale fluid channel structures can be simpler because the fluid dynamics do some of the work, reducing complexity in fabrication and operation.

The curve design, sometimes called “microfluidic drifting”, requires four or five fluid reservoirs to operate [27]. Fluid moves through a 90-degree curve in a microchannel. Sample fluid is located on the inside of the curve and buffer fluid on the outside. As the fast-moving fluids travel around the bend, secondary flow occurs, drawing the sample fluid out horizontally. Additional buffer fluid envelopes the vertically focused sample stream from the sides, performing horizontal focusing and completing the 3DHDF.

The contraction-expansion array design (CEA) works in a similar manner, inducing secondary flow in the form of Dean Vortex generation [28]. Rather than a curve or bend, the CEA consists of a chain of narrow and wide fluid channel sections that cause the fluid to contract and expand. The sample fluid is located on the inside of the narrow contraction regions. When it reaches the expansion region, the sample fluid is horizontally drawn out before being contracted again. The pressure change between the regions induces the secondary flow and the effect compounds. Unlike the Curve design, the CEA completes 3DHDF with no additional buffer fluid for horizontal focusing. In this case, three fluid reservoirs are required for operation.

The last of the single-layer, vortex generator designs discussed here is the microstructure stream-sculpting design [29]. More commonly known as micropillars, the structures are located within the volume of the microfluidic channel in the path of the already horizontally focused sample stream. When the sample stream comes in contact with the micropillars, its path is redirected around the structures and secondary flow occurs. The effect is much like that of the CEA where the contraction and expansion caused by the row of pillars has a compounding effect, thereby sculpting the stream from a vertical stripe to a horizontal ellipse sheathed by buffer fluid. This design requires three to four fluid reservoirs, as the sample stream must be horizontally focused prior to its contact with the microstructures.

Note that these designs are more typical at the 100-μm scale or larger. When shrunk to the 10-μm scale, the fluidic resistance of the small cross-section fluid channels dramatically increases and the flow velocities that are required to induce the secondary flow focusing effect become impractical due to the massive microfluidic channel backpressures [17]. Thus, low fluid velocity is required and the existing single-layer designs become impossible to implement. This means that we cannot rely on secondary flow to do the work of focusing. We must rely on a sheathing or “volumetric flow ratio” design that can be operated at low velocity and in small cross-section channels.

We present a 3DHDF design requiring just one layer of sacrificial material. The low-pressure chamber design, or pit, requires four fluid ports to operate and it promises improved device yield over multi-layer designs. Moreover, it operates by pressure driven volumetric flow ratio, enabling low velocity focusing in channels with dimensions around 10 µm. We will present computer simulations, fabrication processes, and optical characterization of the focusing completed in microscale channels. Fluorescent beads in a flowing aqueous solution are used to characterize the design and demonstrate the enhanced detection capabilities of the system. 

## 2. Materials and Methods 

### 2.1. Design Concept

We have previously reported 3DHDF at the 10-μm scale while using a volumetric flow ratio technique [30]. However, complexity in the fabrication and operation remained. The design required three consecutive layers of sacrificial SU-8 photoresist that formed the fluid channel volume. The dimension steps from layer to layer introduced crevices in the silicon dioxide overlayers that were used to form the walls of the channel leading to structurally weak devices. Moreover, the design required six fluid reservoirs to operate, taking up large amounts of chip real-estate and making operation challenging. A two-layer design was also implemented, improving the yield and decreasing operation complexity by using four fluid reservoirs [31]. However, the multi-layer structure remained less than optimal and resulted in low device yield.

Quantifying fabrication complexity is challenging, as it includes processing time and difficulty. For example, multi-layer photoresist designs are extremely difficult in that subsequent layers must be perfectly processed on the first try, whereas single-layer designs allow for layer stripping and repatterning. Additionally, each layer attempt requires between three and six hours. Moreover, multi-layer designs prove to be less robust, resulting in low device yields. Every parallel processed device can become simultaneously damaged near the end of the multi-week fabrication process, requiring complete device remanufacturing. Moving to a single-layer design greatly simplifies the fluid volume formation process. 

**Figure 2:**
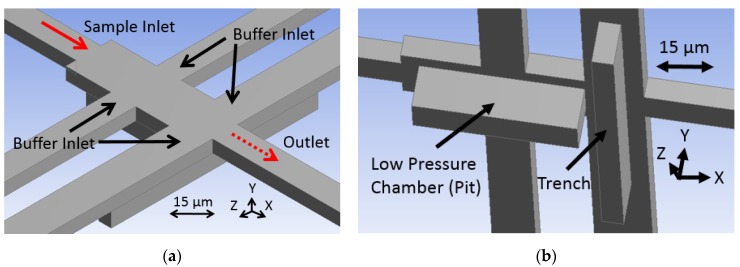
Schematics show (**a**) an above oblique view of the focusing junction describing the operation principle in which sample flow is shown in red, buffer flow in black, and outlet flow in dashed red, and (**b**) a beneath oblique view showing the etched features.

The schematics presented in Figure 2a,b show the fluid junction region of the more easily fabricated, more robust design introduced by this paper. It is formed with one layer of sacrificial photoresist and requires four fluid reservoirs to operate by pressure-driven volumetric flow ratio. The key fluid features that cause the fluid focusing are a pit and trench pair. These are etched into the substrate before a sacrificial material is deposited, covered, and etched out leaving the channels hollow. The pit refers to a large low-pressure chamber that is in-line with the sample stream flow path that the stream comes in contact with, causing it to drop and flatten out. Simultaneously, buffer fluid is injected above the sample stream to occupy the volume above it before both rise out of the pit and they are met from all sides by additional buffer fluid provided by the trench feature. The combined fluid stream exits the junction through the outlet as a 3D focused sample stream with a form factor that is similar to the cross-section of the outlet channel and ready for optical interrogation. The fluids are typically drawn through the chip by applying vacuum pressure to the outlet fluid port, but fluid can alternatively be pushed through by applying pressure to the inlet ports.

**Figure 3:**
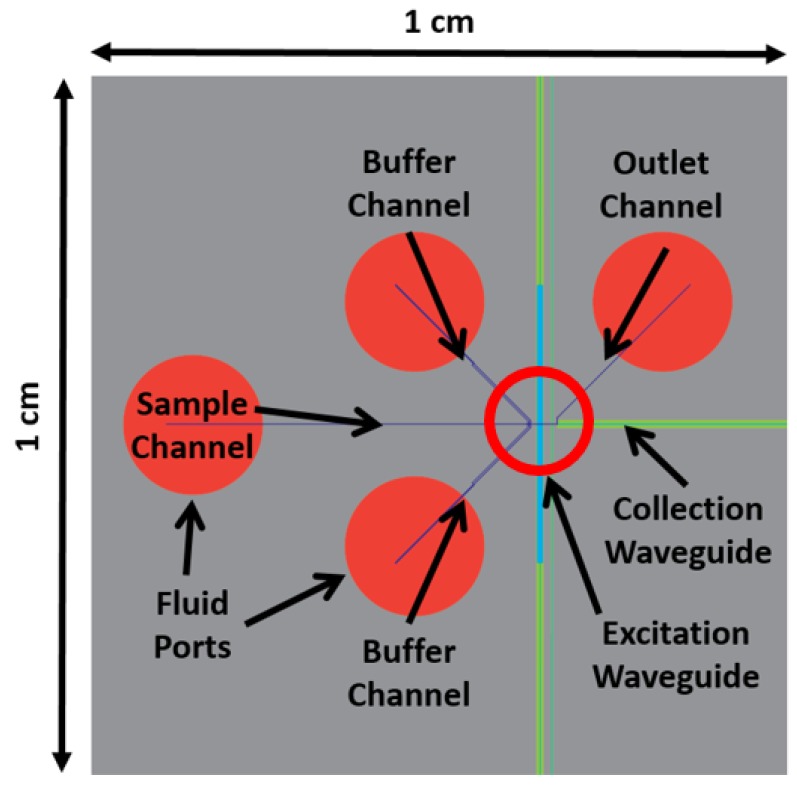
The focusing junction integrates into the optofluidic chip design near the center of the chip, indicated with the red ring, drawn from a top view.

Figure 3 shows a top down view of the optofluidic chip with the 3DHDF element integrated. The silicon-based chip is 1 × 1 cm^2^. Four fluid reservoirs manage the sample, buffer, and waste fluid on the chip. The fluid on the chip flows from left to right, with the buffer fluid coming in contact with the sample fluid near the center of the chip at the focusing junction. The fluid focusing occurs before the liquid-core waveguide fluid channel is intersected by solid-core waveguides that carry light for fluorescence excitation and signal collection. This intersection point is called the excitation region, or excitation volume when referring to the fluid.

### 2.2. Channel Dimension Determination

The fluid channel dimensions between the focusing junction at chip center and the fluid ports were determined by solving a pair of volumetric flow ratios (*Q/Q*) while using the known hydrodynamic relation *P* = *QR* or *Q* = *P/R*, where *P* is pressure, *Q* is volumetric flow rate, and *R* is fluid resistance. This relation is derived from Newton’s second law, to the Navier–Stokes equations, to the Hagen–Poiseuille relation. The relation assumes incompressible laminar Newtonian fluid flow through a circular pipe. The hydraulic radius is substituted to apply the relation to a rectangular duct. The pair of volumetric flow ratios solved were of the buffer fluid flow and sample fluid flow, and of the buffer fluid flow and the outlet fluid flow. The pressure drop was chosen to be approximately equal between the ports and the fluid focusing junction, thus canceling out and leaving just the *R* terms. The *R* terms were expanded into the Hagen–Poiseuille form, the constants canceled out, and the hydraulic radius substituted, leaving

(1) $$\frac{Q_{2}}{Q_{1}}=\frac{L_{1}}{L_{2}}\frac{R_{H}{}_{2}}{R_{H}{}_{1}}=\frac{L_{1}}{L_{2}}\frac{{\left(2w_{1}+2h_{1}\right)}^{4}}{w_{1}^{4}h_{1}^{4}}\frac{w_{2}^{4}h_{2}^{4}}{{\left(2w_{2}+2h_{2}\right)}^{4}}$$

*L* is fluid channel length, *w* is fluid channel width, and *h* is fluid channel height. Subscript 2 indicates terms relating to the buffer fluid channel and subscript 1 indicates terms that relate to the sample (or outlet) fluid channel. This equation was input into an online graphical calculator, called Desmos (San Francisco, CA, USA), which allowed for us to keep track of parameters and view the solution visually. Each *Q* ratio value was input manually as calculated from the input velocities and cross-sectional areas of the inlets from the CAD model (*Q = VA)*. The ratio was subtracted from both sides of the equation to create the slope intercept form for graphing. One of the dimension variables in the equation was replaced with the variable *x* to act as the solution. The equation was multiplied by a large number in order to cause the graphed line to appear nearly vertical for ease of dimension determination. The variable values and solution variable were adjusted until the solutions were deemed as acceptable for fabrication tolerances.

The height of the microchannels is 6 μm. The etch depth is 12 μm. The sample inlet channel is 12 μm wide and 4700 μm long. The buffer channels are 2500 μm long and 20 μm wide, but they split in two halfway from the port to the junction; the pit directed channel is 7 μm wide and the trench channel 12 μm wide. The outlet channel is 12 μm wide and the length is 2500 μm. Determining the dimensions based on volumetric flow ratio means the device can be operated by negative pressure at the outlet over a range of flow velocities.

### 2.3. Modeling

The design that is outlined in Figure 2 was developed in the computational fluid dynamics software Fluent (ANSYS, Canonsburg, PA, USA). The design was meant to be integrated with previously developed optofluidic channels, so it was constrained in outlet channel cross sectional dimensions of 12 μm × 6 μm [32]. The pit and trench etch features were designed to have the same depth of 12 μm so as to simplify etching processes during fabrication. Note that the pit determines the vertical dimension of the focused sample stream and the trench determines the vertical location of the focused sample stream at the outlet.

**Figure 4:**
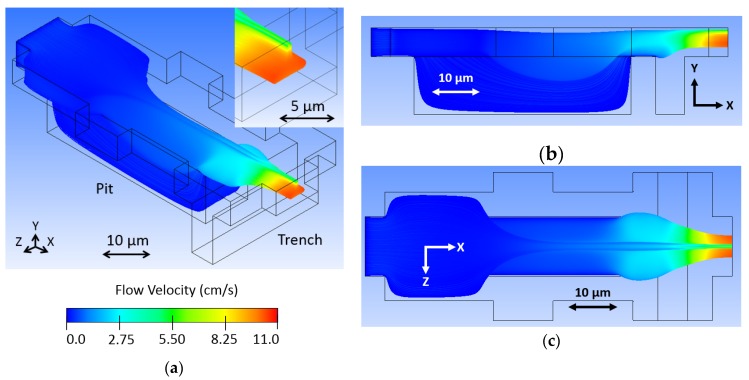
Sample fluid flows in the X direction of the fluid junction model in an (**a**) oblique view, (**b**) side view, and (**c**) top view, where buffer fluid is transparent and sample fluid is colored from dark blue to red, representing the range of flow velocities from slow to fast. A uniformly distributed particle concentration at the sample inlet becomes a focused particle distribution at the outlet.

The model, as visualized in Fluent, is shown in Figure 4, where sample fluid begins as uniformly distributed particle traces and the buffer fluid is invisible. Sample fluid flows in the X direction from left to right, first expanding laterally before coming in contact with the low-pressure chamber, or pit. At this location, buffer fluid travels in the Z direction toward the center and it comes in contact with the sample fluid, pressing it down and occupying the top space of the channel. The sample stream rises up out of the low-pressure chamber before coming in contact with additional buffer fluid in the trench, also traveling in the Z direction toward the center. This raises the sample stream from the bottom toward the center of the channel, effectively 3D focusing the sample stream, as seen at the outlet (see the inset of Figure 4a). Note that the sample stream exhibits a nose shaped cross-section, where a majority of particle streams are predicted to be contained in the center of the channel vertically, but a small portion extend over the main body toward the top of the channel. This is a result of optimizing the design geometry while using large finite elements. This caused streamlines to end abruptly inside the model, resulting in an incomplete stream profile that appeared to be better focused. Increasing the element resolution (decreasing element size) overcame this issue and resulted in the complete profile seen in the figure. The model in Figure 4 used a body fitted cartesian mesh method with half micron element size, resulting in 182,222 finite elements. Although the design was optimized, tooled, and fabricated before the incomplete profile was discovered, it will be shown that the device operation could be adapted for more optimal stream focusing.

The color visible in the Fluent model represents fluid velocity of the sample stream. Blue shows slower flow streams and red faster. The sample stream flow velocity increases through the fluid junction, because the buffer fluid occupies volume and both exit the junction together. The fluid must flow faster to move the combined fluids out with the same fluid volume flow rate with which the fluids are entering the junction. 

The simulation was performed with sample and buffer inlet velocities of 1 cm/s and an outlet pressure of 0 Pascals. Fluid dynamics of the microchannels dictate laminar flow, thus the shortened channels in the simulation. We expect focused flow to propagate down the channel for optical interrogation. However, if the particles are diffusing outside of the focused stream, this behavior would not be accounted for by the fluid dynamic software, as seen in hundred-micron channel length models.

That the modeling conditions do not resemble the experimental conditions was a conscious choice, because the channel length is more than two orders of magnitude greater than the cross-section. Modeling the entire length of the fluid channels from inlet reservoirs to outlet reservoirs, especially given the negligible inertial focusing effects, is unhelpful. Instead, the focusing junction was modeled with the inlet velocities and outlet pressure and the channel dimensions on chip were determined by volumetric flow ratio to achieve the modeled velocities. This was expected to result in the predicted hydrodynamic effect by using negative pressure-driven flow. The experimental results show focused stream flow velocities on the same order of magnitude as predicted.

**Figure 5:**
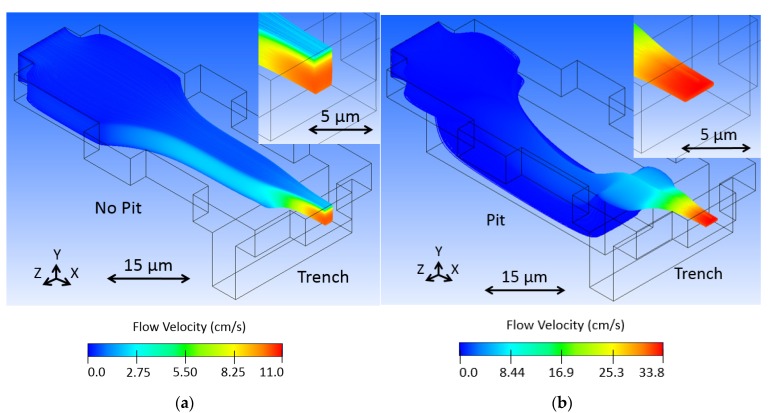
Variations were modeled to show how fluid channel shape and function alter the hydrodynamic focusing effect. (**a**) No pit leaves the horizontally focused sample stream near the top of the channel, a sort of 2.5D HDF. (**b**) Increasing buffer flow by 2.5 times better shapes the sample stream and removes the bridge of the nose shape.

We modeled the design with some variations to portray how certain changes to the design affect the hydrodynamic focusing. First, we removed the pit feature in Figure 5a to show how it functions for vertical focusing. Without the pit, strong horizontal focusing occurs as the buffer fluid squeezes the sample stream from the sides. Subsequently, the trench feature raises up and further squeezes the sample stream. The resulting sample stream is what might be called 2.5D focused, as it is, in fact, limited in position in the fluid channel, but only focused on three sides instead of four. The pit is necessary to move the sample stream downward as buffer fluid fills the top of the channel, as described earlier.

We investigated how controlling fluid flow might enable us to overcome the suboptimal nose shaped sample stream seen in Figure 4. By increasing the buffer fluid input velocity, we expected a greater amount of vertical focusing as a result of the sample stream being more fully covered in the pit region. Figure 5b shows two and a half times the velocity, 2.5 cm/s, where the bridge of the nose shape disappears. Note that sample flow velocity increases with an increasing buffer flow, as all of the fluid entering the junction must leave through one port at the same volumetric flow rate that it enters at the other five ports combined. The increase in buffer fluid limits the amount of sample fluid that can be processed through the device per time, which should be considered in any time-sensitive optofluidic application, for instance, when screening an entire volume of liquid, which contains particles of interest. However, the increase in fluid velocity might compensate for this.

The current design could be adapted to induce optimal flows by increasing the volumetric flow ratio of the buffer fluid. This could be performed with fluid inlet velocity control rather than negative pressure driven flow at the outlet, or the straightforward addition of back pressure at the buffer inlets while using gravity or some other means of positive pressure to push more buffer fluid through [33]. However, high operational vacuum pressures dominate such gravity-driven back pressure effects, as will be discussed.

### 2.4. Fabrication

**Figure 6:**
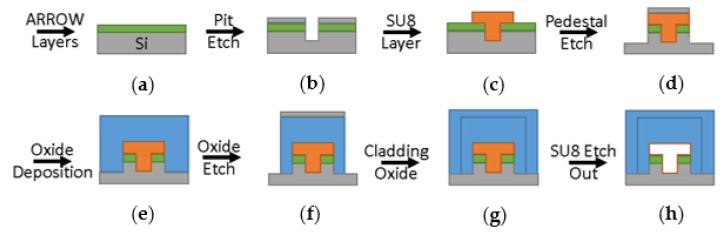
The fabrication flow chart shows cross-section drawing on critical silicon-based microfabrication processes used to build the device, with silicon substrate in gray, thin metal etch masks in gray, anti-resonant reflecting optical waveguide structure (ARROW) layers in green, SU-8 photoresist in orange, and PECVD oxide in blue. (**a**) ARROW layers enable liquid-core waveguiding, and (**b**) the low-pressure chamber (pit) and trench features are etched before (**c**) SU-8 photoresist forms the fluid volume. (**d**) A pedestal helps to improve yield and isolate the optics and (**e**) PECVD oxide forms the walls. (**f**) The liquid-core and solid-core waveguides are etched and (**g**) cladding PECVD oxide protects it all. (**h**) Finally, acid removes the sacrificial SU-8 photoresist, leaving the channels hollow.

The optofluidic device was fabricated in a class 10 cleanroom while using standard silicon-based microfabrication processes. Figure 6 shows side view drawings of the critical process steps. First, commercial layers of silicon dioxide and tantalum pentoxide were deposited on a blank silicon wafer at thicknesses of 265 nm and 102 nm, respectively. These dielectric layers form the anti-resonant reflecting optical waveguide structure (ARROW) required for waveguiding in a low refractive index medium such as a liquid. Next, chrome was deposited on the wafer and patterned with AZ 3330 photoresist and chrome etchant to form a stop etch feature for a later step. Next, nickel was deposited on the wafer and again patterned with AZ 3330 and nickel etchant. The features were ICP-RIE etched, forming the low-pressure chamber (pit) and trench to 12 μm deep. Here, the sacrificial material of SU-8 10 photoresist was deposited and patterned at 3000 rpm spin speed and 30 s exposure time, filling the etched features and forming the liquid-core channel volume. Note that the 6 μm tall resist overlaps all of the edges of the etched features by about 5 μm to ensure coverage and allow for some fabrication tolerance. Next, a self-aligned pedestal was created by depositing and developing AZ 4620 off the top of the SU-8 resist, then depositing nickel before removing the remaining resist with acetone. This liftoff step leaves the nickel mask on top of the defined pedestal to act as a dry etch mask for a 6 μm pedestal. The oxide was deposited next as low stress, high refractive index plasma-enhanced chemical vapor deposited (PECVD) silicon dioxide. Nickel was deposited again, patterned, and the oxide was then etched to form the liquid-core walls and solid-core bodies. An additional low stress, yet low refractive index PECVD silicon dioxide layer was deposited as an environmental barrier [34]. Finally, the sacrificial material was exposed at the ends of the channels with buffered hydrofluoric acid while using AZ 4620 as a mask, and the SU-8 was etched out with a mixture of hydrogen peroxide and sulfuric acid at a ratio of 3:2.

**Figure 7:**
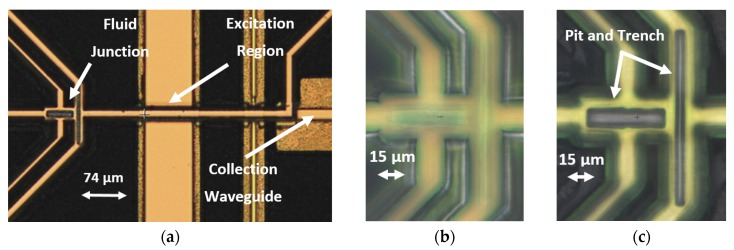
Optical three-dimensional (3D) profilometer images of a completed device center show (**a**) the liquid-core focusing junction and channel and the intersecting solid-core waveguides as well as (**b**) the top of the focusing junction oxide and (**c**) the inside of the focusing junction showing the etch features.

The critical features of a completed device are seen from the top down in Figure 7, showing the fluid focusing junction, as well as the liquid-core channel being intersected by solid-core waveguides. Note how the solid-core features in Figure 7a rest on a pedestal wider than themselves, while the pedestal of the liquid-core channels is not visible from the top, because it only lies directly underneath these features. The pedestal strengthens the hollow channels as it moves the crevice of the oxide all the way to the etched substrate surface. Figure 7b,c show the top of the focusing junction oxide as well as the pit and trench features inside the focusing junction.

## 3. Results

### 3.1. Experimental Methods

**Figure 8:**
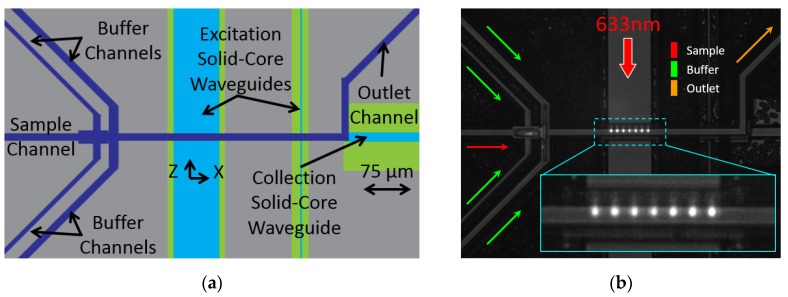
(**a**) Top down schematic of the center of the optofluidic chip. Fluid flows from left to right in the dark blue liquid-core channels, passing through the focusing junction before interacting with the light delivered by the light blue solid-core waveguides. (**b**) Photograph of on-chip multi-mode interference excitation waveguide (MMI) multi-spot excitation scheme using fluorescent dye and DI water.

The sample and buffer fluid flow from left to right and hydrodynamic focusing occurs in the fluid junction shortly before the optical excitation region as seen from top down in Figure 8, which correlates to the center of the chip layout found in Figure 3. It is at this excitation region where the liquid-core waveguide fluid channel and the solid-core MMI waveguide intersect and the light-matter interaction occurs by multi-spot excitation (see Figure 8b), which enables the direct determination of the particles’ flow speed. The solid-core waveguides are oriented perpendicular to the edge of the chip, with the excitation waveguides vertical and the collection waveguide horizontal. Note how the liquid-core waveguide guides fluorescent signal emission to the collection waveguide, which is coupled to the liquid-core and guides the signal to the chip edge, where it can be collected and analyzed.

Three-dimensional focusing was confirmed and the diffusion of the focused sample stream in buffer fluid was characterized in a previously published design similar to this one [31]. The present design shares liquid-core waveguide dimensions (12 μm × 6 μm cross-section), functional method (buffer fluid focusing), and operational flow rates (~3 cm/s), as well as modeling parameters, fabrication processes and materials, and testing methods. Good agreement between predicted and actual streamlines found in the previous design can thus translate to the current design. Moreover, the inertial focusing effects are found to be negligible by calculating the maximum Reynolds number to be less than one. The low diffusivity of the fluorescent beads in conjunction with the predicted laminar flow stream, previous experimentation, and calculated low Reynolds number make us confident that particles remain in hydrodynamically focused streams. Focusing is indicated by an analysis of signal distributions rather than repeating previous experiments.

The previous experiments were performed with Cy5 fluorescent dye and water which were drawn through the device with negative pressure and illuminated with a 633 nm wavelength laser coupled to the chip through a single mode optical fiber (Newport, Irvine, CA, USA, FS-V). The photonic signal passed through a penta-bandpass optical filter (Semrock, Rochester, NY, USA, FF01-440/521/607/694/809-25) before it was detected by a Time-Correlated Single Photon Counting (TCSPC) avalanche photodiode (APD) (PicoQuant, Berlin, Germany, TimeHarp 260 Nano) and analyzed using our event detector scripts developed in Python and available on github (San Francisco, CA, USA) (github.com/vganjali/EventDetector). It utilizes continuous wavelet transform (CWT) with custom made wavelets to enhance the signal in both time and scale domains, followed by the threshold method to localized events accurately in time and scale. Scale information is designed to match with the Δt value that was related to subsequent bright spots for a given multi-peak signal (representing the multi-spot illumination pattern shown in Figure 8b) and it is used as a means to determine the velocity of the detected fluorescent beads [35]. If using diffusible samples such as dye is desired, design constraints should be considered. This includes locating the focusing junction nearer the excitation region and flowing the sample fluid at the highest possible velocity (negative vacuum pressure) to mitigate the lateral spreading of the focused stream. 

**Figure 9:**
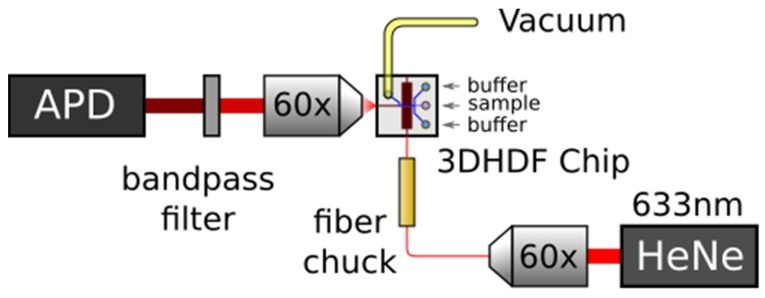
The test system includes the optofluidic chip connected to a vacuum for generating negative pressure to pull sample and buffer fluid through. A 633 nm laser is coupled to the excitation waveguide through an optical fiber to elicit fluorescent photon emission which is collected and detected by an avalanche photodiode (APD) for analysis.

The same apparatus, as shown in Figure 9, was used for evaluating the present design, but here we used 200 nm dark red fluorescent beads (FluoSpheres^TM^ Carboxylate-Modified Microspheres from Invitrogen^TM^, Carlsbad, CA, USA) diluted to 10^7^/mL concentration to elicit discrete signal peaks for analysis. The laser light is coupled to the multi-mode interference excitation waveguide (MMI), which was designed to generate a 75 μm long multi-spot illumination pattern at the intersecting liquid-core waveguide, as seen in Figure 8b.

The lateral spreading of molecules in a focused stream is approximately proportional to the square root of diffusivity. The beads have a reported diffusivity of about 2 × 10^−8^ cm^2^/s, two orders of magnitude lower than the dye [36,37]. Two-hundred nm beads should exhibit little lateral spreading due to diffusion and be useful in characterizing the detection enhancement of the 3DHDF design. This means that the beads are representative of molecules with similar or even higher diffusivity, such as DNA, which are of interest in optofluidic interrogation.

### 3.2. Fluorescent Signal Coefficients of Variance

**Figure 10:**
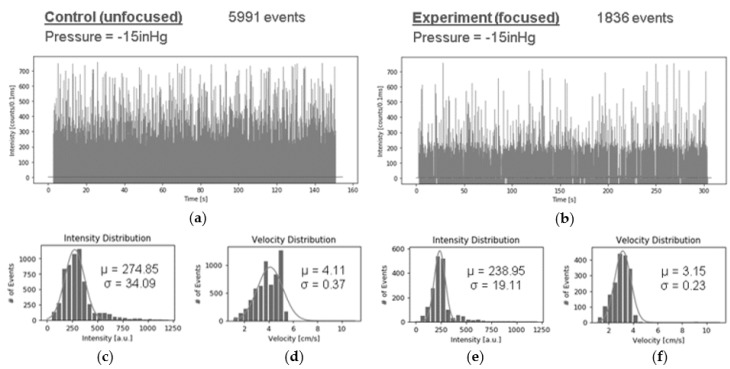
Signal intensity traces in counts per 0.1 ms were collected for (**a**) unfocused operation over 150 s and (**b**) focused operation over 300 s. (**c**–**f**) Histogram distributions for signal intensity and fluid velocity show more consistent signal with three-dimensional hydrodynamic focusing (3DHDF).

The effect of 3DHDF on fluorescent signal quality was measured by first filling all of the fluid channels with a fluorescent bead solution as a control test. In this case, there is no effective focusing of a particle stream because the entire cross section of a fluid channel is filled with particles. Measured fluorescence for this case was then compared to the case, where the sample fluid channel was filled with solution containing beads and the buffer channels with water. In both cases, fluid was drawn through the optofluidic channels with negative pressure at the outlet and illuminated with multiple spots of 633 nm laser light through the solid-core MMI waveguide. An avalanche photodiode at the chip edge collected the fluorescent signal captured orthogonally in the liquid-core waveguide and a computer reported the intensity in counts per 0.1 ms, as observed in the traces in Figure 10. The control intensity trace in Figure 10a represents a uniformly bead filled fluid channel and Figure 10b represents a focused bead stream that was sheathed by buffer fluid. 

These plots show the measured optical intensity versus time, with intensity spikes representing fluorescing beads passing through an excitation point. When comparing the control trace to the experiment trace we see an almost sevenfold decrease in events captured per time. This is likely due to the buffer fluid in the 3DHDF case occupying a large fraction of the channel volume and reducing the number of beads passing through the channel per time. Figure 10c–f show histogram distributions of both signal intensity and fluid velocity for the control and 3DHDF cases. For both the intensity and velocity distributions, we see a narrowing due to hydrodynamic focusing, which is expected.

An improvement in detection performance can be represented by the coefficient of variance (CV) for the measured signal. This is calculated by dividing the standard deviation of the signal intensity by the mean of the distribution. A smaller CV represents a narrowing of the distribution or an increase in mean. They both indicate enhanced detection of optofluidic sensors promised by HDF. The data found in Figure 10c–f were used to generate CV values for characterizing detection enhancement. The resulting signal intensity CVs are 0.12 and 0.08 for the control and experiment, respectively, a decrease of 33%, which is one way to quantify the increase in signal uniformity that is afforded by 3DHDF. The calculated CV for the fluid velocity distributions are 0.09 and 0.07 for the control and experiment respectively, a decrease of 22%, again with 3DHDF showing improved uniformity. The data were obtained while using −15 inHg vacuum-pressure-driven flow to overcome the reservoir effects while maintaining high signal intensity, as will be discussed shortly.

### 3.3. Pressure Dependent Detection Enhancement

**Figure 11:**
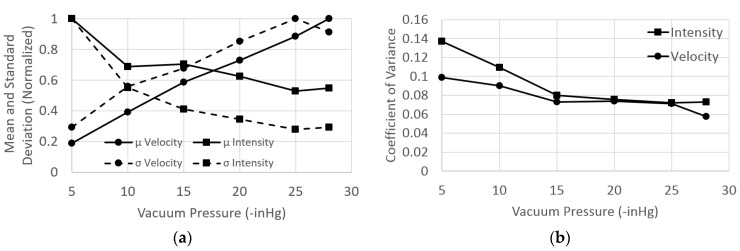
(**a**) With increasing vacuum pressure, the mean (μ) and standard deviation (σ) of the velocity increase while those of the intensity decrease (**b**) The coefficient of variance (CV) decreases with increasing vacuum pressure for both signal intensity and particle velocity before flattening out. Back pressure widens the focused sample stream at low operational pressures but is dominated at higher pressures.

We wanted to test how the mean and standard deviation of signal traces that were collected at various vacuum pressures would relate. Increasing the vacuum pressure results in a greater pulling force on the fluid through the channels, and thus higher fluid velocities and a lower chance of detecting all emitted photons. This is related to the time resolution and dead time of the detector. Fluorescence happens on the nanosecond scale, which means the beads should have enough time to be excited and emit fluorescent signal, but the detector is unable to detect all of the received photons in the short time interval that the fluorophore spends in the excitation volume. The result is lower signal intensities. We changed the pressure in increments of 5 from −5 inHg to −25 inHg as well as −28 inHg, the limit of the vacuum system. Figure 11a shows the normalized mean and standard deviation values of the signal intensity and fluid velocity. As expected, the velocity increased and the intensity decreased with increasing vacuum pressure magnitude. The mean fluid velocity ranged from approximately 1 cm/s to 5.4 cm/s. 

Figure 11b plots the coefficient of variance for intensity and velocity for each vacuum pressure. The trend observed is a result of reservoir effects. This is seen as higher CV values at low operational vacuum pressures that drop with increasing vacuum pressure before flattening out. The sample channel has higher fluidic resistance than the buffer channels, due to the sample channel being narrower than the buffer channels. This means the buffer channels consumed reservoir fluid volume faster than the sample channel, which results in an increase in back pressure on the sample fluid due to the greater height of fluid in the column [33]. During experimentation, an unbalance of volumetric flow ratio occurred, resulting in a less tightly focused sample stream (higher CV). The reservoir effect is dominant at low pressures, but it becomes negligible at higher vacuum pressures, being seen as the flattening of the CV line. The flat portion matches expectations as the device is designed to operate over a range of velocities with negligible focusing change. 

We concluded that a minimum of −15 inHg vacuum pressure should be used to overcome the reservoir effects that were observed in the testing of the device. This finding, in conjunction with the signal intensity data found in Figure 11a, led us to perform device characterization at −15 inHg vacuum pressure (as shown in Figure 10) to overcome the observed reservoir effects while maintaining the greatest signal intensity. Negative pressure driven flow schemes used for optofluidic detection should similarly balance signal variance with signal intensity to achieve optimal detection schemes.

We compared the device yield of the silicon-based microchips in over 300 total devices. The present design showed an average of 45% yield when compared to the average 29% yield of the previous design, a 1.56 times improvement in device yield. We attribute this yield improvement to the robust design while using a single-layer of photoresist to form the fluid channels. 

## 4. Conclusions

A 10-μm scale, single layer 3D hydrodynamic focusing design was implemented, which improved device yield by 1.56 times over previous work. The low-pressure chamber, or pit, is the key feature that induces 3D focusing using buffer fluid that is delivered by negative pressure-driven flow, controlling the position of target particles in the fluid channel during fluorescent signal generation. This mitigates the disadvantageous natural phenomena that are present in microfluidic channels by narrowing signal intensity and flow velocity distributions and constraining target particles to highest collection efficiency regions. It does this while using just four fluid reservoirs. The concept, modeling, fabrication, and testing were outlined. Fluorescent beads representing molecules of interest were analyzed to find 33% more consistent signal with focusing. The reservoir effects were observed at low operational vacuum pressures and an optimal detection scheme was used to maximize signal consistency while maintaining high signal intensity. Signal intensity and flow velocity coefficients of variance matched expectations by decreasing between the control and the experiment, indicating successful focusing and detection enhancement. Further improvement and optimization in signal intensity can be achieved by aligning focused particle streams with the optical excitation mode center and with collection modes in the liquid-core waveguide. This could be achieved with the current geometry while using independent fluid inlet controls to flow additional buffer fluid volume. A gravity driven back-pressure method would be ineffective at higher operational vacuum pressures as the effect becomes dominated. Alternatively, the pit approach could be redesigned to efficiently operate with negative pressure-driven flow.
